# Impact of Bone-Grafting Materials on the Rate of Orthodontic Tooth Movement: A Systematic Review

**DOI:** 10.7759/cureus.44535

**Published:** 2023-09-01

**Authors:** Bassam Alalola, Ali Asiri, Ibraheem Binmoghaiseeb, Waleed Baharoon, Yazeed Alrassi, Bandar Alanizy, Hesham Alsayari

**Affiliations:** 1 Preventive Dental Science, College of Dentistry, King Saud bin Abdulaziz University for Health Sciences, Riyadh, SAU; 2 Research and Development, King Abdullah International Medical Research Center, Riyadh, SAU; 3 Dental Department, Ministry of National Guard Health Affairs, Riyadh, SAU; 4 Research Department of Epidemiology & Public Health, Institute of Epidemiology and Health Care, University College London, London, GBR

**Keywords:** alloplast, xenograft, allograft, bone graft, rate of orthodontic tooth movement

## Abstract

Orthodontists may encounter patients with alveolar bony defects, which are often treated with various bone-grafting materials. The effects of different bone-grafting materials on orthodontic tooth movement (OTM) are of concern to orthodontists. Therefore, we aimed to evaluate the current status of the literature that reports on the effects of different bone-grafting materials on OTM in terms of the rate and side effects. An electronic search of the PubMed and Scopus databases and Google Scholar was performed. Two reviewers independently conducted the screening process using COVIDENCE™, and a third reviewer resolved any conflicts. SYRCLE’s (Systematic Review Centre for Laboratory Animal Experimentation's) risk-of-bias tool for animal studies was utilized to assess the quality of the included studies. Out of 457 initial titles, 11 studies were finally included for data extraction. All of the included studies were animal experiments, and none of them were considered to have a low risk of bias. The included studies had varied results. However, a general tendency existed, whereby OTM in surgically treated areas with no bone grafting presented the highest OTM rate. In cases where a bone graft was used, xenografts revealed the highest OTM rate, followed by alloplasts. Lastly, the use of allografts resulted in the slowest OTM rates. The most common side effect was root resorption. In conclusion, there is a lack of high-quality evidence regarding the effects of bone-grafting materials on OTM rate. Due to the lack of human subjects, RCTs, and the heterogeneity of subjects in the current literature, the impact of bone-grafting materials on OTM deserves further investigations using more rigorous scientific methodologies.

## Introduction and background

Orthodontic patients, with pre-existing alveolar defects or unfavorable ridges, lead to progressive alveolar destruction post-ortho treatment [1-4]. Evidence-based studies reveal that guided bone regeneration is the preferred procedure to treat these defects and ensures optimal periodontal health conducive to orthodontic tooth movement (OTM) [5,6]. Favorable alveolar bone morphology is essential for any desired orthodontic movement [7]. Nevertheless, moving the teeth into existing alveolar bony defects may result in fenestrations or dehiscence [8]. Compromised periodontal status can inhibit active orthodontic tooth movement or lead to root resorption and tooth loss. In clinical conditions where the patients have chronic periodontitis, congenital alveolar clefts, or constant occlusal trauma, an orthodontic force can induce further alveolar bone loss [9]. In addition, a narrow alveolar ridge is observed in individuals of ectomorphic phenotype limiting the range of orthodontic tooth movement [10]. Hence, when orthodontic management is planned with associated pathologic conditions bone augmentation is necessary [11]. However, the effects of different bone-grafting materials on OTM are of great concern to orthodontists.

The biological range of orthodontic movement has been broadened by alveolar cortical surface bone grafting facilitated by periodontally accelerated osteogenic orthodontics [12]. Desirable and minimal tooth movement is reported through primary woven bone with pre-orthodontic bone grafting [11]. An Ideal pre-orthodontic bone grafting material should be safe and enable desired OTM [13]. Bone grafts are used to induce bone formation and remodeling to compensate for bone defects, and they are categorized into autogenous and non-autogenous grafts [14,15]. Autogenous bone grafting is a procedure wherein the bone is harvested from the same individual to augment bone defects; this is considered the gold standard approach in the treatment of bone defects [15,16]. The Iliac crest graft is the most common donor site for extra-oral grafts in oral and maxillofacial surgery [17]. However, the morbidity associated with iliac crest harvest and complications reported to rates up to 49%, including damage to blood vessels and nerves, joint disruption, fractures, subluxation, hernia, and delayed iliac abscess* *[17]. Also, mandibular symphysis, anterior mandibular ramus, and maxillary tuberosity have been used as sources of intraoral donor sites for autogenous bone grafting [15,16,18]. The amicable characteristics of the autogenous bone grafts include being osteoconductive and osteoinductive potential, abundance in spongy bone in proximity to alveolar bone structure, and regeneration of the covering periodontium favoring the orthodontic teeth movement [19]. However, limitations like the rate of graft resorption, which varies from 22% up to 33% must be taken into consideration. Additionally, cost, size mismatch, and additional auto graft harvest surgery have to be considered, which gives way for substitute graft materials [20].

Non-autogenous bone grafts can be allografts, xenografts, or alloplasts [15,21]. Allografts use bone graft materials obtained from other members of the same species [21]. Decalcified freeze-dried bone allogeneic and freeze-dried bone allogeneic grafts are the most commonly utilized for pre-orthodontic allografts but with questionable osseo-inductive potency [19]. Xenografts such as Bio-Oss (Geistlich Pharma AG, Wolhusen, Switzerland) and Gen-Tech (Baumer SA, Mogi Mirim, SP, Brazil) are used as bone grafts that are acquired from a donor of a different species to the recipient [22]. With xenografts, orthodontic treatment is often impaired and root resorption is evident as a result of orthodontic stress on the root surface when alveolar bone augmentation is performed [23,24]. Alloplasts like Nanobone (Artoss GmbH, Rostock, Germany) and BoneCeramic (Institut Straumann AG, Basel, Switzerland) are synthetically created bone grafts that promote pre-orthodontic alveolar bone augmentation [25]. However, root resorption and gingival invagination make allografts unfavorable for pre-orthodontic bone grafting [11].

Orthodontic tooth movement is a physical phenomenon that relies on Newton’s laws and bone tissue remodeling [26]. Different force magnitudes and the direction and duration of the applied forces produce bone remodeling and changes in tooth position [26]. This phenomenon is infamously slow and attempts to accelerate it have been the focus of orthodontic literature for the past years by utilizing a wide variety of interventions [27,28]. However, the effect of bone grafts in repaired alveolar defects on the rate of OTM is underexplored.

In recent years, orthodontic treatment and bone grafting have gained popularity, especially in the adult population. Thus, a patient with a grafted bone will have an improved scope of orthodontic treatment. Therefore, researchers have started to explore whether bone-grafted areas interact differently with OTM and whether different bone grafts have different effects on OTM, the grafted area, and the teeth [29]. Over the last decade, several studies have investigated OTM in bone-grafted areas. However, various studies have demonstrated conflicting results [19,23,30,31]. Due to these different conclusions and the range of novel bone-grafting materials used, we aimed to evaluate and discuss the effects of the pre-orthodontic bone-grafting materials on the rate of OTM and associated side effects.

## Review

Methodology

The present systematic review is according to Preferred Reporting Items for Systematic Reviews and Meta-Analyses (PRISMA) guidelines. This systematic review was registered with PROSPERO (CRD42032403760). The research question for this review was “What is the impact of different bone-grafting materials on the rate of orthodontic tooth movement?”.

An electronic search was conducted using PubMed, EMBASE, Scopus, and Google Scholar, considering publications pertaining to the topic up to May 2023. The data obtained from the search results was exported to a systematic review management software tool (COVIDENCE™). A prompt screening of the titles, abstracts, and full-text articles after removing the duplicates was done. The search terms included the following keywords: “bone transplantation”, “alveolar bone grafting”, “bone graft”, “allograft”, “xenograft”, “alloplast”, “autogenous bone graft”, “nonautogenous bone graft”, “xenogenic graft”, “tooth movement techniques”, “orthodontic tooth movement”, “rate of tooth movement”, “speed of tooth movement”, and “tooth movement”. Additionally, the gray literature was also searched for pertinent literature. However, a manual search of the bibliographies of the selected studies yielded relevant studies.

The inclusion criteria included articles written in English providing information on OTM after alveolar bone grafting in terms of rate, speed, time, or side effects in both human and animal studies. Studies that consisted of subjects with a congenital bony defect, tooth eruption or impaction, OTM after orthognathic surgery, uncontrolled medical conditions, lack of a control group, subjects on bisphosphonates, and Wilckodontics studies were excluded. Also, no time/date restrictions were applied in the search strategy.

The research team selected and reviewed the studies using a three-step process using COVIDENCE™. Two researchers (A.A. and I.B.) independently screened and reviewed the titles, abstracts, and full texts according to the eligibility criteria. The researchers resolved conflicts through discussions and consensus. In the case that the two researchers did not reach a consensus, they consulted a third researcher (B.A.).

Quality and bias risk assessment was performed by utilizing the Systematic Review Centre for Laboratory Animal Experimentation’s risk-of-bias tool (SYRCLE’s RoB) for animal studies because all the studies that met the eligibility criteria used animal subjects. SYRCLE’s RoB tool includes 10 domains: sequence generation, baseline characteristics, allocation concealment, random housing, performance blinding, random outcome assessment, detection blinding, incomplete outcome data, selective outcome reporting, and other bias sources. 10 points need to be scored for each study. In each domain, multiple questions called signaling questions guided the authors in determining the RoB for each study. Each point was scored as YES (low bias risk), NO (high bias risk), or UNCLEAR (unclear bias risk) by answering the signaling questions. Two reviewers (A.A. and I.B.) independently applied the RoB for the included studies, and a third reviewer (B.A.) addressed any conflicts. Moreover, Microsoft Excel 365 was used to tabulate the extracted data into two tables, one of which shows the OTM details and the other shows the characteristics of the animals and bone grafts. In Table 1, we grouped the studies based on the authors, publication year, animal model, sex, number of subjects, subjects’ age, defect location, and type of graft used. Table 2 provides a detailed summary of the included studies regarding OTM, wherein we recorded the following parameters: OTM type, force used, OTM timing, OTM duration, analysis method, the average OTM amount, standard deviation, significance level, and side effects.

**Table 1:** Animal model and bone graft characteristics. E, experimental group; C, control group; EL, experimental with laser group; b-TCP, beta-tricalcium phosphate; NM, not mentioned.

Authors, Year	Animal Model	Gender	N	Age	Defect Location	Type of Graft Material—Grouping
Klein et al. (2020) [19]	C57BL mice	male	30	6–7 weeks	maxillary left 1st molar	A- Alloplast (b-TCP): 10 subjects
						B- Allograft (mice femurs and tibias): 10 subjects
						C- Control: 10 subjects
Klein et al. (2019) [13]	C57BL mice	male	44	6–7 weeks	maxillary left 1st molar	A- Xenograft (Bio-Oss): 22 subjects
						B- Control: 22 subjects
Machibya et al. (2018) [30]	Beagle dogs	male	6	18 months	all 1st premolars	A- Xenografts (Bio-Oss): 8 sites
						B- Alloplast (b-TCP): 8 sites
						C- Control: 8 sites
Ru et al. (2018) [23]	Sprague Dawley rats	male	60	5 weeks	maxillary left 1st molar	A- Alloplast (BoneCeramic): 20 subjects
						B- Xenograft (BioOss): 20 subjects
						C- Control: 20 subjects
Ru et al. (2016) [24]	Sprague Dawley rats	male	60	5 weeks	maxillary left 1st molar	A- Alloplast (BoneCeramic): 20 subjects
						B- Xenograft (Bio-Oss): 20 subjects
						C- Control: 20 subjects
Kim et al. (2015) [32]	Beagle dogs	male	10	18–24 months	maxillary 1st premolar	A- Xenograft (Bio-Oss with OrthoBlast): 8 sites
						B- Graft with laser 8 sites
						C- Control: 4 sites
Ahn et al. (2014) [33]	Beagle dogs	male	12	18–24 months	maxillary 1st premolar	A- Xenograft (Bio-Oss with OrthoBlast): 6 subjects
						B- Control: 6 subjects
Seifi & Ghoraishian. (2012) [31]	German race dogs	male	3	13 ± 1 months	all 3rd premolars	A- Allograft (CenoBone^®^): 6 sites
						B- Control: 6 sites
Oltramari et al. (2007) [34]	Minipigs (BR-1)	male	6	12 months	all 4th deciduous molars and the germs of the 4th premolars	A- Xenograft (Gen-Tech): 12 sites
						B- Control: 12 sites
Araújo et al. (2001) [29]	Beagle dogs	NM	5	~1 year	mandibular 1st, 2nd, and 4th premolars	A- Xenograft (Bio-Oss): 5 sites
						B- Control: 5 sites
Sheats et al. (1991) [35]	Cats	male	12	NM	mesial to the mandibular 1st premolars	A- Alloplast (b-TCP): 12 sites
						B- Control: 12 sites

**Table 2:** The OTM rate OTM, orthodontic tooth movement; E, experimental group; C, control group; EL, experimental with laser group; b-TCP, beta-tricalcium phosphate; NM, not mentioned

Authors, Year	OTM Type, Force Used	OTM Timing	OTM Duration	Analysis Method	Avr. OTM Amount	Standard Deviation	Significant Difference	Side Effects
Klein et al. (2020) [19]	Bodily, 10 g	4 weeks	3 weeks	micro-CT	1- Control = 921.7 um	1- Control group = 48.9 um	No	NM
					2- Alloplast = 707.3 um	2- Alloplast = 30.6 um		
					3- Allograft = 648.3 um	3- Allograft = 31.6 um		
Klein et al. (2019) [13]	Bodily, 10 g	4 weeks	A- 2 weeks = 11 E, 11 C subjects	micro-CT	1- Control 3 weeks = 836.72 um	1- Control 3 weeks = 130.831	Yes	NM
					2- Xenograft 3 weeks = 550.36 um	2- Xenograft 3 weeks = 101.52		
			B- 3 weeks = 11 E, 11 C subjects		3- Control 2 weeks = 480.81 um	3- Control 2 weeks = 128.60		
					4- Xenograft 2 weeks = 371.7 um	4- Xenograft 2 weeks = 76.30		
Machibya et al. (2018) [30]	Bodily, 150 g	A- 4 weeks, 4 E1, 4 E2, 4 C sites	8 Weeks	Intraoral digital caliper and CT	1- Xenograft 4 weeks = 4.08 mm	1- Xenograft 4 weeks = 0.57 mm	Yes	NM
					2- Xenograft 8 weeks = 4.35 mm	2- Xenograft 8 weeks = 0.83 mm		
					3- Alloplast 4 weeks = 4.50 mm	3- Alloplast 4 weeks = 0.36 mm		
		B- 8 weeks, 4 E1, 4 E2, 4 C sites			4- Alloplast 8 weeks = 5.02 mm	4- Alloplast 8 weeks = 1.10 mm		
					5- Control 4 weeks = 5.14 mm	5- Control 4 weeks = 0.18 mm		
					6- Control 8 weeks = 4.71 mm	6- Control 8 weeks = 0.74 mm		
Ru et al. (2018) [23]	Bodily, 10 g	4 weeks	4 weeks	micro-CT	1- Control: 0.9 mm	NM	Yes	Root resorption
					2- Xenograft: 0.7 mm			
					3- Alloplast: 0.65 mm			
Ru et al. (2016) [24]	Bodily, 10 g	4 weeks	4 weeks	micro-CT	1- Control = 1 mm	NM	Yes	Root resorption
					2- Alloplast = 0.8 mm			
					3- Xenograft = 0.8 mm			
Kim et al. (2015) [32]	Bodily, 100 g	A- immediately 4 E, 4 EL, 4 C sites	6 weeks	Stone models and micro-CT	1- Control = 1.28 mm	1- Control = 0.13 mm	Yes	NM
					2- Xenograft immediate = 3.44 mm	2- Xenograft immediate = 1.25 mm		
					3- Xenograft 2 weeks = 2.42 mm	3- Xenograft 2 weeks = 0.84 mm		
		B- 2 weeks, 4 E, 4 EL sites			4- Xenograft with laser immediate = 1.59 mm	4- Xenograft—laser immediate = 0.23 mm		
					5- Xenograft 2 weeks with laser = 1.06 mm	5- Xenograft—laser 2 weeks = 0.35 mm		
Ahn et al. (2014) [33]	Bodily, 100 g	A- immediately = 2 E, 2 C subjects	6 weeks	Stone models and micro-CT	1- Control immediate = 2.30 mm	1- Control immediate = 0.07 mm	Yes	NM
					2- Control 2 weeks = 3.51 mm	2- Control 2 weeks = 0.07 mm		
		B- 2 weeks = 2 E, 2 C subjects			3- Control 12 weeks = 1.18 mm	3- Control 12 weeks = 0.04 mm		
					4- Xenograft immediate = 3.44 mm	4- Xenograft immediate = 0.07 mm		
		C- 12 weeks = 2 E, 2 C subjects			5- Xenograft 2 weeks = 2.42 mm	5- Xenograft 2 weeks = 0.14 mm		
					6- Xenograft 12 weeks = 1.75 mm	6- Xenograft 12 weeks = 0.09 mm		
Seifi & Ghoraishian. (2012) [31]	Bodily, NM	Immediately	8 weeks	Intraoral boley gauge caliper	1- Allograft = 3.7 mm	1- Allograft = 1.83 mm	Yes	NM
					2- Control = 2.7 mm	2- Control = 1.7 mm		
Oltramari et al. (2007) [34]	Bodily, 4.5 N	12 weeks	17 weeks	NM	1- Xenograft = 4 mm	NM	No	Root resorption
					2- Control = 4 mm			
Araújo et al. (2001) [29]	Bodily, 30–50 cN	12 weeks	NM	NM	1- Xenograft = 3.85 mm	1- Xenograft = 0.57 mm	NM	Root resorption
					2- Control = 3.37 mm	2- Control = 0.45 mm		
Sheats et al. (1991) [35]	Bodily, 100 g	6 weeks	9 weeks	Intraoral dial Vernier caliper	1- Alloplast = 1.12–3.47 mm	NM	No	NM
					2- Control = 0.76–3.01 mm			

Results

**Figure 1:**
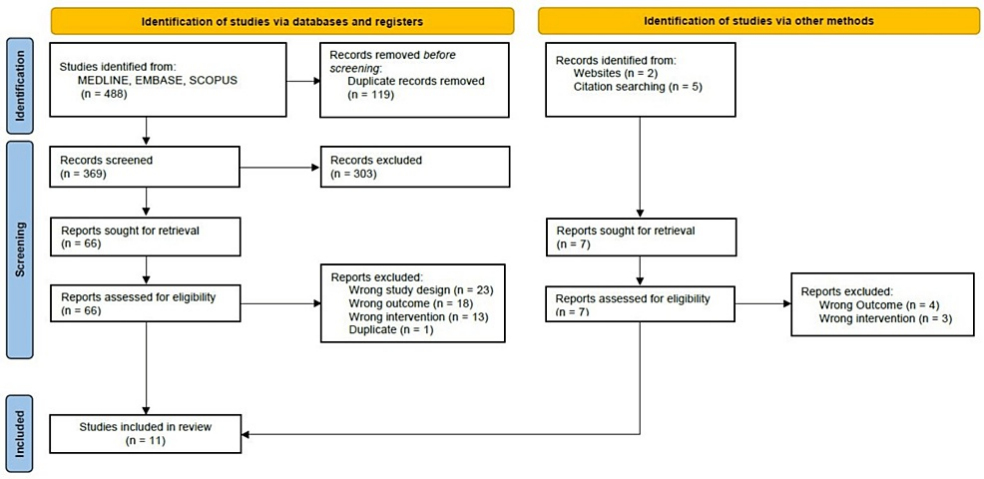
PRISMA flow diagram showing the screening and inclusion process PRISMA, Preferred Reporting Items for Systematic Reviews and Meta-Analyses

The present study identified a total of 488 articles, including seven articles that we manually retrieved. One hundred duplicate articles were eliminated using COVIDENCE™. After title and abstract screening, 303 articles were excluded, which resulted in 66 articles that were eligible for full-text screening. During the full-text screening, we excluded 55 studies for the following reasons: wrong study design, wrong outcomes, wrong intervention, and one duplicate, which resulted in 11 studies being included in the systematic review. The PRISMA flow diagram presents a summary of the screening process (Figure 1). Table 1 describes the selected studies’ characteristics, and Table 2 provides a detailed summary of OTM where reported.

All the subjects in the included studies were animals. The authors of the included studies used six different kinds of animals and a total of 248 animals (120 rats, 74 mice, 33 beagle dogs, 12 cats, six minipigs, and three German racing dogs). Dogs were the most tested subjects, as they were the test subjects in five studies [29-33], followed by rodents, namely, rats and mice, in four studies [13,19,23,24], and minipigs and cats in one study each [34,35]. The animals’ ages ranged from five weeks to two years (Table 1). In all the studies, the authors surgically created defects in the experimental groups in premolar or molar areas (Table 1).

**Figure 2:**
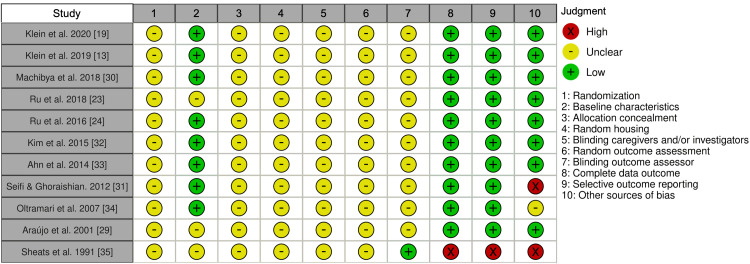
Individual SYRCLE bias risk assessment SYRCLE, Systematic Review Centre for Laboratory Animal Experimentation

The control group had the fastest OTM rate in most studies, followed by those that received a xenograft, then an alloplast, and lastly, an allograft (Table 2). The authors of eight studies did not mention any side effects, whereas the authors of the other three mentioned root resorption as a side effect in all groups (Table 2) [23,24,34]. However, only one study mentioned the resorption severity, noting that it was the strongest in the control group and weakest in the alloplast (BoneCeramic) group [30]. Among the 11 evaluated studies, the individual risk of bias was mostly unclear (Figure 2).

Discussion

This systematic review tried to answer the research question, which was “What is the impact of the different pre-orthodontic bone-grafting materials on the rate of OTM and associated side effects?” Results showed that there was a general tendency regarding OTM into surgically treated areas with no bone-grafting results in the highest rate of OTM. In cases where a bone graft was used, xenografts revealed the highest rate of OTM followed by alloplasts. After that, allografts resulted in the slowest rates of OTM. The most common side effect was root resorption. Despite the inter-relationship between orthodontics and alveolar defects, scholars have conducted only limited investigations focusing on this topic [30]. Furthermore, there is a scarcity of human randomized clinical trials to investigate the effect of bone-grafting materials on consequent OTM rates. However, researchers have conducted several experimental animal studies and have reported a wide heterogeneity regarding study subjects, study designs, and analysis modes, which affects the translation of these results into clinical practice. The authors of several case reports studied the possibility of OTM through a bone-grafted area. Unfortunately, they did not investigate the effects on the OTM rate, lacked a study control, or studied only the periodontal effects of such procedures [11,36-38].

The experimental model varied across the studies, with some including 14 rodents such as mice and rats [13,19,23,24], which can be obtained in large numbers for a low cost and can therefore be used to achieve more accurate results through the use of large study samples. The authors of most studies utilized dogs, specifically beagle dogs [29-33]. Some authors also used minipigs and cats [34,35].

Heterogeneity was observed in terms of the anatomical location and characteristics of the bone defects, bone augmentation materials, and tooth movement directions, which made it difficult for us to make accurate comparisons. OTM timing has a critical role in the OTM rate. The authors of the chosen studies used different time points to evaluate OTM, ranging from immediate OTM to three months after grafting. The timing of OTM initiation is a key point in determining the fastest OTM through grafted bone. Klein et al. suggested that the optimal timing is four weeks post-regeneration with bone grafts, as full osseous healing is achieved and remains stable at six weeks [13]. However, the authors of most studies did not explain their reasoning for their chosen OTM timing.

The authors of the included studies used the following types of trade market grafts: alloplast (beta-tricalcium phosphate, b-TCP) [19,30,35], alloplast (BoneCeramic) [23,24], xenograft (Bio-Oss) [13,19,23,24,29], xenograft (Bio-Oss with OrthoBlast (IsoTis OrthoBiologics, Irvine, California) [32,33], and xenograft (Gen-Tech) [34]. Seifi M et al. used an allograft (CenoBone®, Tissue Regeneration Corporation, Kish Island, Iran) and allograft (mice femurs and tibia) [19,31]. Overall, the control groups that did not include grafted defects experienced the highest OTM rate in six studies, with differences in the OTM timing [13,19,23,24,30,33]. Xenografts were the most presented bone substitution materials, used in almost eight of the studies. Furthermore, the OTM rate of the xenograft group was slower than that of the control group in five of the studies [13,23,30,33] and was faster in two studies [29,32]. In one of the studies, Oltramari et al. (2007) found that the OTM rate of the xenograft group was equal to that of the control group [34].

Alloplasts were the second most presented bone substitution material, as the authors of five studies utilized them [13,23,24,30,35]. Two studies found that it was the slowest material [27,30], whereas the authors of one study found that it was the fastest [35]. In Klein et al. (2020), the alloplast group had a slower OTM rate than the control group and a faster OTM rate than the allograft group [19]. This is similar to the report of Machibya et al. (2018), who found that the alloplast group had a slower OTM rate than the control group and a faster OTM rate than the xenograft group [30]. The authors of two studies used allografts; Klein et al. (2020) found that it was the slowest material, and Ghoraishian et al. and Seifi et al. (2012) found that it was the fastest [13,31].

In regards to timing, four of the studies [13,19,23,24] concluded that the control group had the fastest OTM rate when initiated after four weeks of grafting [33]. They concluded that OTM initiation two weeks after grafting resulted in the OTM rate of the control group being faster than when the OTM was immediately initiated or initiated after 12 weeks. The xenograft group had the fastest OTM rate when the OTM was started immediately or after two weeks [32,33]. They found that the OTM through the alloplastic bone graft material was faster if initiated after eight weeks [30]. The authors of a recently published scoping review focused on animal model studies that investigated OTM timing in defects augmented with synthetic bone scaffolds, and they reported similar conclusions in relation to OTM timing and side effects [39]. Additionally, Tsai et al. 2021 reported that OTM timing should coincide with the stage of woven bone formation during the regional acceleratory phenomenon, which will lead to shorter orthodontic treatment times [39]. A key factor in determining the rate and timing of OTM is the biodegradation of grafting materials [16]. The variable degradation rate of different bone-grafting materials is largely attributed to the composition of scaffold biomaterials [40]. Therefore, biodegradability is required to provide stable bone matrix formation and reduce root damage [33]. Nevertheless, initiating OTM immediately before graft degradation may result in a fast OTM rate, yet the involved mechanisms require further investigation.

This systematic review described and summarized the effects of different pre-orthodontic bone-grafting materials on the rate of OTM and associated side effects. Researchers may rely on this paper's results to have an overview and prepare for well-designed human clinical trials that may help clinicians shorten the orthodontic treatment periods.

Limitations

The present study included only studies on animal subjects that matched the eligibility criteria. However, due to the differences between each species and humans in terms of anatomy and physiology, there are certain limitations. However, researchers controlled the subjects’ parameters and uniform study environment to achieve constant results. Generally, the quality of the available literature was low because of the perceived unclear-to-high bias risk (Figure 2) in the included articles. Unclear randomization, allocation concealment, random housing, unclear blinding of caregivers and outcome assessors, the loss of data and selective reporting, and other bias sources could have confounded the results. Therefore, the results of this systematic review should be interpreted with caution. Furthermore, the heterogeneity of the study designs, including participant characteristics and interventions, creates an obstacle to outcome comparisons and evaluation.

## Conclusions

In conclusion, this systematic review summarized all studies that reported positive tooth movement. The fastest OTM was reported in cases with no bone grafting, and the slowest was in cases with an allograft bone graft. The OTM rate is affected by the variable degradation rate of different bone-grafting materials and the formation of a new bone matrix. Moreover, few studies reported that root resorption is a side effect of moving teeth through bone grafts. The quality of the literature was concerning, and the bias risk was mostly unclear to high. Future well-designed studies conducted on animals and humans are necessary in order to translate the outcomes of future research into clinical practice.

## References

[1] Talic NF (2011). Adverse effects of orthodontic treatment: a clinical perspective. Saudi Dent J.

[2] Sebbar M, Abidine Z, Laslami N, Bentahar Z (2015). Periodontal Health and Orthodontics. Periodontal Health and Orthodontics. InTech.

[3] Mathews DP, Kokich VG (1997). Managing treatment for the orthodontic patient with periodontal problems. Semin Orthod.

[4] Jafer M, Crutzen R, Moafa I, van den Borne B (2021). What do dentists and dental students think of oral cancer and its control and prevention strategies? A qualitative study in Jazan Dental School. J Cancer Educ.

[5] Mayer Mayer, T.; Basdra, E.K.; Komposch, G.; Staehle, H.J H.J (1994). Localized alveolar ridge augmentation before orthodontic treatment. A case report. Int J Oral Maxillofac Surg.

[6] Diedrich PR (1996). Guided tissue regeneration associated with orthodontic therapy. Semin Orthod.

[7] Machado GL (2015). CBCT imaging - a boon to orthodontics. Saudi Dent J.

[8] Miao Y, Chang YC, Tanna N (2022). Impact of frontier development of alveolar bone grafting on orthodontic tooth movement. Front Bioeng Biotechnol.

[9] Seifeldin SA (2016). Is alveolar cleft reconstruction still controversial? (Review of literature). Saudi Dent J.

[10] Alfallaj H (2020). Pre-prosthetic orthodontics. Saudi Dent J.

[11] Ghezzi C, Masiero S, Silvestri M, Zanotti G, Rasperini G (2008). Orthodontic treatment of periodontally involved teeth after tissue regeneration. Int J Periodontics Restorative Dent.

[12] Tokhtah HA, Alhadlaq AM (2022). Utility of injectable bisphosphonates in enhancing orthodontic retention in a goat model: a split-mouth study. Saudi Dent J.

[13] Klein Y, Fleissig O, Stabholz A, Chaushu S, Polak D (2019). Bone regeneration with bovine bone impairs orthodontic tooth movement despite proper osseous wound healing in a novel mouse model. J Periodontol.

[14] Zhao R, Yang R, Cooper PR, Khurshid Z, Shavandi A, Ratnayake J (2021). Bone grafts and substitutes in dentistry: a review of current trends and developments. Molecules.

[15] Kumar P, Vinitha B, Fathima G (2013). Bone grafts in dentistry. J Pharm Bioallied Sci.

[16] Lu J, Wang Z, Zhang H (2022). Bone graft materials for alveolar bone defects in orthodontic tooth movement. Tissue Eng Part B Rev.

[17] Almaiman M, Al-Bargi HH, Manson P (2013). Complication of anterior iliac bone graft harvesting in 372 adult patients from May 2006 to May 2011 and a literature review. Craniomaxillofac Trauma Reconstr.

[18] Mellonig JT (1992). Autogenous and allogeneic bone grafts in periodontal therapy. Crit Rev Oral Biol Med.

[19] Klein Y, Kunthawong N, Fleissig O, Casap N, Polak D, Chaushu S (2020). The impact of alloplast and allograft on bone homeostasis: orthodontic tooth movement into regenerated bone. J Periodontol.

[20] Fourcade C, Lesclous P, Guiol J (2019). Assignment of autogenous bone grafts for reconstruction of the alveolar ridge before implant placement. J Oral Med Oral Surg.

[21] Kao ST, Scott DD (2007). A review of bone substitutes. Oral Maxillofac Surg Clin North Am.

[22] Cardoso GB, Tondon A, Maia LR, Cunha MR, Zavaglia CA, Kaunas RR (2019). In vivo approach of calcium deficient hydroxyapatite filler as bone induction factor. Mater Sci Eng C Mater Biol Appl.

[23] Ru N, Liu SS, Bai Y, Li S, Liu Y, Zhou G (2018). Microarchitecture and biomechanical evaluation of BoneCeramic grafted alveolar defects during tooth movement in rat. Cleft Palate Craniofac J.

[24] Ru N, Liu SS, Bai Y, Li S, Liu Y, Wei X (2016). BoneCeramic graft regenerates alveolar defects but slows orthodontic tooth movement with less root resorption. Am J Orthod Dentofacial Orthop.

[25] de Almeida Malzoni CM, Gonçalves V, Possari J, Junior EM (2022). The use of 3D ceramic block graft compared with autogenous block graft for rehabilitation of the atrophic maxilla: a randomized controlled clinical trial. Trials.

[26] Jeon HH, Teixeira H, Tsai A (2021). Mechanistic insight into orthodontic tooth movement based on animal studies: a critical review. J Clin Med.

[27] Fu T, Liu S, Zhao H, Cao M, Zhang R (2019). Effectiveness and safety of minimally invasive orthodontic tooth movement acceleration: a systematic review and meta-analysis. J Dent Res.

[28] Gkantidis N, Mistakidis I, Kouskoura T, Pandis N (2014). Effectiveness of non-conventional methods for accelerated orthodontic tooth movement: a systematic review and meta-analysis. J Dent.

[29] Araújo MG, Carmagnola D, Berglundh T, Thilander B, Lindhe J (2001). Orthodontic movement in bone defects augmented with Bio-Oss. An experimental study in dogs. J Clin Periodontol.

[30] Machibya FM, Zhuang Y, Guo W, You D, Lin S, Wu D, Chen J (2018). Effects of bone regeneration materials and tooth movement timing on canine experimental orthodontic treatment. Angle Orthod.

[31] Seifi M, Ghoraishian SA (2012). Determination of orthodontic tooth movement and tissue reaction following demineralized freeze-dried bone allograft grafting intervention. Dent Res J (Isfahan).

[32] Kim KA, Choi EK, Ohe JY, Ahn HW, Kim SJ (2015). Effect of low-level laser therapy on orthodontic tooth movement into bone-grafted alveolar defects. Am J Orthod Dentofacial Orthop.

[33] Ahn HW, Ohe JY, Lee SH, Park YG, Kim SJ (2014). Timing of force application affects the rate of tooth movement into surgical alveolar defects with grafts in beagles. Am J Orthod Dentofacial Orthop.

[34] Oltramari PV, de Lima Navarro R, Henriques JF (2007). Orthodontic movement in bone defects filled with xenogenic graft: an experimental study in minipigs. Am J Orthod Dentofacial Orthop.

[35] Sheats RD, Strauss RA, Rubenstein LK (1991). Effect of a resorbable bone graft material on orthodontic tooth movement through surgical defects in the cat mandible. J Oral Maxillofac Surg.

[36] Reichert C, Wenghöfer M, Götz W, Jäger A (2011). Pilot study on orthodontic space closure after guided bone regeneration. J Orofac Orthop.

[37] Michelogiannakis D, Makou M, Madianos P, Rossouw E (2017). Orthodontic tooth movement in relation to angular bony defects. Aust Orthod J.

[38] Ruellas AC, Cunha AC, Valadares CV, Tenorio De Sa AP, Ruellas CV (2016). Orthodontic movement of posterior teeth into a corticocancellous bone-block allograft area. J Clin Orthod.

[39] Tsai MH, Megat Abdul Wahab R, Yazid F (2021). Timing of orthodontic tooth movement in bone defects repaired with synthetic scaffolds: a scoping review of animal studies. Arch Oral Biol.

[40] Zhang H, Zhou L, Zhang W (2014). Control of scaffold degradation in tissue engineering: a review. Tissue Eng Part B Rev.

